# A multilevel analysis of social capital and self-reported health: evidence from Seoul, South Korea

**DOI:** 10.1186/1475-9276-11-3

**Published:** 2012-01-26

**Authors:** Sehee Han, Heaseung Kim, Hee-Sun Lee

**Affiliations:** 1 Department of Public Administration, Hanyang University, 17 Haengdang-dong, Seongdong-gu, Seoul, 133-791, South Korea; 2 Graduate School of Public Policy, Hanyang University, 17 Haengdang-dong, Seongdong-gu, Seoul, 133-791, South Korea

**Keywords:** Social capital, self-reported health, multilevel analysis, South Korea, Seoul

## Abstract

**Background:**

This study aims to resolve two limitations of previous studies. First, as only a few studies examining social capital have been conducted in non-western countries, it is inconclusive that the concept, which has been developed in Western societies, applies similarly to an Asian context. Second, this study considers social capital at the individual-level, area-level and cross-levels of interaction and examines its associations with health while simultaneously controlling for various confounders at both the individual-level and area-level, whereas previous studies only considered one of the two levels. The purpose of this study is therefore to examine the associations between social capital and health by using multilevel analysis after controlling for various confounders both at the individual and area-levels (i.e., concentrated disadvantage) in non-western countries.

**Methods:**

We conducted a cross-sectional survey from December 2010 to April 2011 in Seoul, South Korea. The target population included respondents aged 25 years and older who have resided in the same administrative area since 2008. The final sample for this study consisted of 4,730 respondents within all 25 of Seoul's administrative areas.

**Results:**

In our final model, individual-level social capital, including network sources (OR = 1.23; 95% CI = 1.11-1.37) and organizational participation (OR = 2.55; 95% CI = 2.11-3.08) was positively associated with good/very good health. Interestingly, the individual × area organizational participation cross-level interaction was negatively associated with good/very good health (OR = 0.40; 95% CI = 0.32-0.50), indicating that in areas with higher organizational participation, individuals with high organizational participation were less likely to report good/very good health when compared to low organizational participation individuals.

**Conclusion:**

Our study provides evidence that individual-level social capital is associated with self-reported health, even after controlling for both individual and area-level confounders. Although this study did not find significant relationships between area-level organizational participation and self-reported health, this study found the cross-level interaction for social capital. Hence, in areas with lower organizational participation, the probability of reporting good/very good health is higher for individuals with high organizational participation than individuals with low organizational participation. This study, albeit tentatively, suggests that policy makers should focus upon social capital when making policies which aim to enhance one's health.

## Background

It has been widely acknowledged that places where people live are a crucial factor for individual health [1-3]. Previous studies have been focused on various physical aspects of places (e.g., environmental pollution) [4]. Other than these factors, there is a growing body of evidence that social environment such as social capital is crucial for individual health [5-7].

In recent decades social capital has become a popular topic in a variety of research areas [8-16]. Research has examined an association between social capital and well-being [17], health behavior [18], economic development [19], democracy [20], educational achievement [21] and many other phenomena. Thus, it is not surprising that researchers have been paying great attention to an association between social capital and health [22-24]. Although the results vary since social capital is not a single unified concept, scholars generally agree that social capital is an important predictor of health [25-29].

The notion of social capital developed over a long period of time through contributions by a number of theorists [30]. Despite their differences, scholars see social capital as a resource accessed through social relations [31-35]. Additionally, although there has been an ongoing debate as to whether social capital is a collective characteristic or a property of individuals [36], many scholars agree that it could be both [37]. There are other unsolved issues, as well. For example, Portes [8] argues that social capital and its effects should be distinguished. Some scholars [38,39] also argue that social capital should be defined by its sources, the social networks, rather than its effects (e.g., trust). Similarly, Lin [40] sees trust as a consequence of social capital and Lin & Erickson [41] also argue that social capital should be defined as social networks themselves and thus trust should be distinguished from social capital based on a series of researches [42,43]. In this study, we define social capital as resources, the property of both individuals and collectivities, which derived from and could be accessed through various forms of social relations.

To date, there are two major criticisms of prior studies on social capital and health. One major limitation of previous research is that although many studies have been conducted in western societies, only a few studies have been conducted in non-western countries [44]. Studies regarding social capital and its association with health are even sparse in Asian countries than in Western-countries, particularly in South Korea. Hence, it is inconclusive whether a similar empirical relationship between social capital and health could be found when applied to an Asian context. Another criticism is that studies which use multilevel analysis to examine the association between social capital and health often considered an individual-level confounder but did not consider other contextual-level confounders. Hence, not controlling for the influence of relevant environmental factors on health may lead to biased conclusions regarding the effects of social capital on health [45]. Among various environmental factors, this study considers socio-economic inequality of place. Because this factor is well recognized as being associated with health [46-48] and because it can be modified by social capital [49], it needs to be controlled as social capital might be confounded by it.

The primary purpose of this study is to examine the association between social capital and health using multilevel analysis allows for the simultaneous inclusion of different levels of social capital and examining its associations with health. The main question of this study was "What aspects and levels of social capital are associated with health in Seoul, South Korea after controlling for various confounders both at individual and area levels?" It is important to confirm whether health benefits of social capital are individual or collective, as they indicate different strategies to suggest for policy recommendations and improve public health.

## Methods

### Sampling and sample size

We conducted a cross-sectional survey from December, 2010 to April, 2011 in Seoul, South Korea since we were not able to find the secondary dataset which includes the crucial variables for this study and targets the general Seoul citizens. The target population included respondents with ages of 25 years and older who have resided in the same administrative area since 2008, because area effects are not immediate and an individual needs a lengthy period of time to adjust to an area environment. Seoul, South Korea's capital and largest city is comprised of 25 administrative areas with approximately 10.5 million residents (about 4 million households) as of 2010. On average, each administrative area is comprised of about 308,000 of residents age 25 and older (ranging from about 100,000 to about 495,000) [50].

The data collected from 25 administrative areas in Seoul was divided into two strata according to degree of urbanization. One strata included three administrative areas which are wealthier than other administrative areas, and another strata includes the other 22 administrative areas. We used sampling quotas based on the population distribution of official census data, including respondents' gender and age in each strata, to ensure a reasonable representation of Seoul citizens. Household phone numbers were chosen using systematic sampling within each administrative area.

We conducted telephone interviews using a telephone directory as a sampling frame from December, 2010 to March, 2011. Calls were made mainly during the evenings and throughout the day on weekends. We called back two times when a selected number did not answer. If they were not reached after a total of three calls were made, they were regarded as invalid. A total of 3,918 samples of individual data were gathered, with a response rate of 43%.

To reflect the census distribution of population, we additionally used a quota sampling to gather data of respondents aged 50 and above. The fieldwork was conducted from March, 2011 to April, 2011. The mode of data collection was a face-to-face interview conducted by interviewers. Respondents were informed of the aim of the study by the interviewers and then volunteers were interviewed. We gathered 832 respondents who were qualified and agreed to participate in this study and completed questionnaire. A total of 4,750 samples of individual data were obtained. However, we excluded 20 cases with missing values on variables which were needed for the study.

It was possible that a sampling modification may have caused a bias, so we considered controlling for it in our models. We created a dummy variable indicating whether respondents surveyed through a quota sampling (yes = 1, no = 0). We further considered the within-area percentages of respondents surveyed through a quota sampling. These variables were statistically associated with respondents' various characteristics and self-reported health. These results indicate a sampling modification may have caused a bias, so it is problematic combining two-survey data without considering it. Thus, we included these variables in our multilevel models. The final sample for this study consisted of 4,730 respondents within all 25 administrative areas.

### Measures

#### Dependent variable

The dependent variable of this study is respondents' self-reported health status. Respondents were asked to rate their own health status on a five-point scale from "very bad" to "very good" by answering the question "What is your current general health status?" This self-reported health status has proven to be a good predictor of mortality [51] and to be relatively less sensitive to variable wordings of the question [52]. For analysis, this original scale was categorized, with 1 representing good and very good health, and 0 representing fair, bad, and very bad health.

#### Social capital variables

Two variables were used to operationalize the individual-level social capital. Network resources were measured using the Position Generator [53]. This instrument measures an individual's social capital by assessing the extent to which each person can access to different occupations through their social networks. For the current study, we created a position generator that describes the context of Korean society using Choi's occupational prestige scale [54]. Twenty-five occupations from Choi's occupational prestige scale were chosen. When respondents were surveyed, they were asked if they knew of any acquaintances, friends, or family members from a list of 25 occupations. If they knew more than one person that fit a certain position, they were asked to choose the first person they could think of. The aforementioned occupations and prestige scores are shown in Table 1.

**Table 1:** Occupations and occupational prestige scores in position generator

Occupation	Prestige score	Occupation	Prestige score
Doctor	85.6	Officer (armed forces)	65.5
Assemblyman	84.7	Police officer	63.9
Professor	84.2	Employee of a large company	61.9
Owner of a large company	83.1	Nurse	61.3
Lawyer	82.7	Computer programmer	58.5
Local governor,	76.3	Technician	54.7
Clergy	75.9	Skilled worker	49.7
Pharmacist	74.5	Small shop owner	48.4
School teacher	71.9	Salesman	43.2
Producer	71.2	Driver	41.0
Entertainer	71.1	Farmer	40.0
Journalist	70.2	Construction worker	31.7
Small-business owner	70.1		
Variables	Mean (S.D.)	Factor loading	Cronbach's alpha

Network diversity	10.05 (5.90)	0.767	0.81
Upper reachability	81.85 (6.75)	0.927	
Range of prestige score	40.94 (11.81)	0.780	

Eigenvalue	2.05		
Explained variance (%)	0.98		

The primary variables extracted from the Position Generator are network diversity, upper reachability and range of prestige score which are generally used to reflect a variety of aspects of personal networks. Network diversity refers to as the total number of occupations accessed through the respondent's social networks out of a total of 25 occupations (ranging from 0 to 25). Second, upper reachability was operationalized as the uppermost resource a respondent can reach through their social networks (ranging from 0 to 85.6). Third, the range of the prestige score was calculated. This was calculated as the difference between the highest and the lowest prestige scores among accessed people by the respondent (ranging from 0 to 53.9). These three measures have similar meanings but also indicate differences. Two people could have the same network diversity or range of prestige scores in their networks, while one person has access mainly to high-prestige positions and the other has mainly the opposite. Factor analysis of these items resulted in a one-factor solution (eigenvalue = 2.05; all items loaded above 0.76) and Cronbach's alpha for this scale was 0.81. Thus, the factor score was calculated and labeled as "network resources."

Organizational participation was measured by the number of organizations to which the respondent belongs. The list of organizations were as follows: religious groups, political parties, hometown organizations, sports/outdoor clubs, hobby/cultural clubs, labor unions, environmental/animal protection organizations, humanitarian aid/human rights organizations, clan meetings through Jong-Chin Organization (a group composed of people with the same family name and family origin on the paternal line), consumer protection groups, veteran's groups, neighborhood watch or neighborhood improvement associations, alumni associations, parent-teacher associations, and volunteer organizations. Each item was coded which 0 representing "non-member/inactive member" and 1 representing "active member." We chose the label "active member" only for members of organizations, as we defined inactive members as people who only enrolled as a member in an organization but did not participate. Thus, an inactive member is not likely to be affected by the organization or other members, so the inactive membership hardly contributes to formatting and maintaining one's social capital. Each item was added and a dummy variable was created with a score of 0 reflecting "low organizational participation (coded = 0)" and a score from 1 to 10 reflecting "high organizational participation (coded = 1)."

One variable was used as an area-level social capital. The area-level organizational participation variable was constructed by standardizing the proportion of respondents who were classified with high organizational participation at the individual-level organizational participation for each area. The mean score of area-level organizational participation before standardization was 0.47 (SD = 0.21).

#### Area-level confounder

The within-area percentages of persons receiving public assistance, female-headed households with children under poverty line, and of male-headed households with children under poverty line in 2009 were measured as the area-level covariates. These three variables were obtained from the Seoul Statistics website [50]. Factor analysis was conducted and revealed a one-factor solution (eigenvalue = 2.338; all items loaded above 0.730). We named this factor "concentrated disadvantage" and the factor score was used in multilevel analysis. The detail statistics of the concentrated disadvantage scale are shown in Table 2.

**Table 2:** Factor and reliability analysis of concentrated disadvantage scale

Individual items and scale	Mean (S.D.)	Factor loading
**Concentrated disadvantage** **(Cronbach's alpha = 0.852)**	0 (1)	-
% persons receiving public assistance	3.03 (1.05)	0.733
% female single parent households with children	0.54 (0.22)	0.933
% male single parent households with children	0.14 (0.06)	0.964

Eigenvalue	2.338	
Explained variance (%)	77.97	

#### Individual-level confounders

We included several individual-level confounders (i.e., age, gender, perceived social class, employment status, educational attainment, marital status, smoking, and drinking). Age was used as a continuous variable. Gender was dichotomized (1 = male, 0 = female). Respondents' perceived social class was originally measured with a four point scale. We combined low to middle-low categories as low social class (coded = 0) and middle-high to high categories as high social class (coded = 1). Employment status was measured (1 = employed including self-employed and 0 = others). Respondents' educational attainment was measured (1 = high school or below, 2 = college or university equivalent degree, and 3 = graduate school) and 1 = high school or below was used as a reference category. Respondents' marital status was originally measured (1 = single, 2 = married, 3 = widowed, 4 = separated, and 5 = divorced). We combined widowed, separated, and divorced categories and used "single" as a reference category. Smoking behavior was measured by the following question: "On average, about how many cigarettes do you smoke per a day?" with responses ranging from no cigarettes to more than two packs a day. This variable was coded 0 for non-smoking and 1 for daily smoking. Drinking behavior was measured by asking, "How often do you drink alcohol?" on a four-point scale indicating "none at all," "sometimes," "drink often but do not get drunk," and "drink often and often get drunk". We combined "sometimes" and "drink often but do not get drunk" into one category (1 = none at all, 2 = sometimes/drink often but not get drunk, 3 = drink often and often get drunk) and used "none at all" as a reference category.

### Statistical methods

The hierarchical data structure considered in the analysis was comprised of 4,730 individuals nested within 25 administrative areas. We investigated the association between social capital and health using multilevel logistic regression analysis because the dependent variable is dichotomized. Multilevel analysis takes into account the independence violation among individuals in the same cluster and distinguishes between compositional and contextual effects [55]. We conducted multilevel logistic regression analyses with individuals at the first level and administrative areas at the second level to distinguish compositional and contextual relationships between independent variables and self-reported health. This model can be defined as:

$$\text{logit}\left(P_{ij}\right)=\beta_{1}+\beta_{2}X_{ij}+\beta_{3}Z_{j}+\mu_{j}$$

*X_ij _*is a vector of individual characteristics of respondent *i *in administrative area *j *and *Z_j _*denotes a vector of administrative area attributes, *μ_j _*denotes the random part, and logit(*P_ij_*) is a sum of the linear function of the independent variables. We estimated an empty model with no predictors first, as this model provides a baseline for comparing the size of area variations in self-reported health. If the dependent variable did not have area variability, then there was little reason to conduct multilevel models.

Model 1: We included all individual-level variables in the fixed part, including social capital. This model assessed the effect of individual-level variables on self- reported health.

Model 2: This is the same as model 1. However, we further included all area-level variables. This model includes relevant area and individual-level variables in the same models, essentially estimating the health effects of variables at one level while controlling for variables at the other, and vice versa.

Model 3: This is the same as model 2. However, we included the cross-level interaction term for individual and area-level organizational participation.

We also calculated the intra-class correlation (ICC) for each model by using the following formula:

$$ICC=\frac{\sigma^{2}}{\sigma^{2}+3.29}$$

*σ*^2 ^is the variance in area-level. The ICC estimates the proportion of variance that is accounted for by the higher level [56].

Odds ratios were used for interpretation. All statistical procedures were performed using STATA 11.0 (StataCorp, College Station, TX).

## Results

Table 3 shows the descriptive statistics of individual-level variables both for the total sample and stratified sample of self-reported health. After excluding missing data of certain variables, including self-reported health and smoking, the study sample was comprised of 4,370 persons within 25 administrative areas. 38.54% of respondents across all administrative areas reported good/very good health. The relatively low percentages of reporting good/very good health might be derived from the fact that as Koreans became interested in health, it may have caused a high expectation regarding their health status which may decrease in satisfaction with subjective health in turn. Additionally, relatively high percentage of respondents answered on a "fair" category (40.17%). The Pearson correlation coefficient between area-level organizational participation and concentrated disadvantage was -0.129 (data not shown). The crude odds ratios for self-reported health according to each independent variable are shown in Table 4. In the empty model, 37.6% (ICC = 0.376) of the total variation in health could be found at the area-level, and the between area variation is significantly different from zero (p < 0.001), indicating that a multilevel model is required. As we mentioned in the sampling and sample size section, combining two-survey data without a relevant consideration may cause a biased result. Thus, we included a dummy variable indicating whether respondents surveyed through a quota sampling and the within-area percentages of respondents surveyed through a quota sampling. We further considered the responses rates per areas, as it may cause the high ICC and also to reduce a selection. Thus, we included all of it and re-estimated the ICC. The ICC reduces to 0.065. Consequently, we adjusted for these variables in all models (data not shown).

**Table 3:** Descriptive statistics for individual-level variables used in final analysis

Outcome Good/very good health	Yes (n = 1,823, 38.54%)	No (n = 2,907, 61.46%)	Total (n = 4,730)
Age (Mean (S.D.), years)	30.09 (14.36)	52.38 (12.31)	47.26 (14.64)
Gender			
Male	1,031 (56.56%)	1,355 (46.61%)	2,386 (50.44%)
^a ^Female	792 (43.44%)	1,552 (53.39%)	2,344 (49.56%)
Social class			
High	855 (46.90%)	978 (33.64%)	1,833 (38.75%)
^a ^Low	968 (53.10%)	1,929 (66.36%)	2,897 (61.25%)
Employment status			
Employed	1,387 (76.08%)	2,324 (79.94%)	3,711 (78.46%)
^a ^Not employed	436 (23.92%)	583 (20.06%)	1,019 (21.54%)
Educational attainment			
^a ^High school or below	386 (21.17%)	825 (28.38%)	1,211 (25.60%)
College or university equivalent degree	1,000 (54.85%)	1,683 (57.89%)	2,683 (56.72%)
Graduate school	437 (23.97%)	399 (13.73%)	836 (17.67%)
Marital status			
^a ^Single	883 (48.44%)	361 (12.42%)	1,244 (26.30%)
Married	886 (48.60%)	2,230 (76.71%)	3,116 (65.88%)
Widowed/divorced/separated	54 (2.96%)	316 (10.87%)	370 (7.82%)
Smoking			
Yes	545 (29.90%)	591 (20.33%)	1,136 (24.02%)
^a^No	1,278 (70.10%)	2,316 (79.67%)	3,594 (75.98%)
Drinking			
Heavy	85 (4.66%)	261 (8.98%)	346 (7.32%)
Sometimes	1,274 (69.88%)	1,026 (35.29%)	2,300 (48.63%)
^a^No	464 (25.45%)	1,620 (55.73%)	2,084 (44.06%)
Network resources (Mean, (S.D.), factor score)	0.10 (0.85)	-0.64 (1.00)	0 (0.94)
Organizational participation			
High	977 (53.59%)	1,252 (43.07%)	2,229 (47.12%)
Low^a^	846 (46.41%)	1,655 (56.93%)	2,501 (52.88%)

^a ^Reference categories used for multilevel analyses.

**Table 4:** Unadjusted odds ratios (OR) with 95% confidence intervals (95% CI) of good/very good health using multilevel logistic analysis (n = 4,730)

	Unadjusted OR (95% CI)
*Individual-level variables*	
Age	0.95 (0.94-0.96)**
Gender	
Female	Referent
Male	1.59 (1.35-1.88)**
Social class	
Low	Referent
High	1.67 (1.42-1.98)**
Employment status	
Not employed	Referent
Employed	0.64 (0.53-0.79)**
Educational attainment	
High school or below	Referent
College or university equivalent degree	0.44 (0.34-0.56)**
Graduate school	0.79 (0.59-1.04)
Marital status	
Single	Referent
Married	0.23 (0.19-0.28)**
Widowed/divorced/separated	0.02 (0.01-0.04)**
Smoking	
No	Referent
Yes	1.08 (0.90-1.28)
Drinking	
No	Referent
Sometimes	2.29 (1.97-2.74)**
Heavy	0.46 (0.34-0.64)**
Network resources	1.08 (0.99-1.18)
Organizational participation	
Low	Referent
High	2.65(2.25-3.12)**
*Area-level variables*	
Concentrated disadvantage	0.90 (0.51-1.60)
Organizational participation	0.63 (0.36-1.10)

*p < 0.05; **p < 0.001.

Table 5 presents the results of the multilevel analyses for health in the order in which they were conducted. The first model (Model 1) included all of the individual-level variables. This model shows that all of the individual-level variables are significantly associated with people's self-reported health. As expected, age is negatively associated with health. The likelihood of reporting good/very good health decreases about 2% per year (OR = 0.98; 95% CI = 0.97-0.99). Male respondents are more likely to report good/very good health than female respondents (OR = 1.73; 95% CI = 1.41-2.11). Compared to unemployed respondents, employed respondents are less likely to report good/very good health (OR = 0.62; 95% CI = 0.49-0.79). Respondents with a college or university equivalent degree (OR = 0.28; 95% CI = 0.21-0.37) and graduate degree (OR = 0.60; 95% CI = 0.43-0.83) are all less likely to report good/very good health than respondents with educational attainment of high school or below. Compared to single respondents, widowed/divorced/separated (OR = 0.02; 95% CI = 0.01-0.03) and married respondents (OR = 0.27; 95% CI = 0.20-0.36) are, on average, less likely to report good/very good health. Not surprisingly, respondents who smoke are less likely to report good/very good health than respondents who do not (OR = 0.64; 95% CI = 0.47-0.89). People who moderately drink are more likely to report good/very good health (OR = 1.59; 95% CI = 1.27-1.99) and heavy drinkers are less likely to report good/very health (OR = 0.43; 95% CI = 0.24-0.75) compared to non-drinkers. Respondents with 1-SD higher on network sources (OR = 1.18; 95% CI = 1.06-1.31) were associated with a significantly higher odds of reporting good/very good health. Respondents with a high level of organizational participation are more likely to report good/very good health than respondents with a low level of organizational participation (OR = 2.03; 95% CI = 1.58-2.60). In Model 2, we further included all the area-level variables in the model. Both area-level variables were unassociated with self-reported health. At the individual-level, employment status was no longer statistically associated with self-reported health. Apart from a slight change in coefficients, the relationship between other individual-level variables and self-reported health remain as significant as in Model 1. Our final model (Model 3) included a cross-level interaction term between individual-level organizational participation and area-level organizational participation. The individual × area organizational participation was negatively associated with health (OR = 0.40; 95% CI = 0.32-0.50). Thus, in areas with lower organizational participation, the probability of reporting good/very good health is higher for individuals with high organizational participation than individuals with low organizational participation. However, this relationship between individual organizational participation and health is reversed in areas with high organizational participation. Additionally, age was no longer statistically significant at the 5% level.

**Table 5:** Multilevel logistic analysis for self-reported health (odds ratios and 95% confidence interval in parentheses; n = 4,730)

Fixed effects	Self-rated health (good/very good health)		
	
	Model 1	Model 2	Model 3
*Individual level variables*			
Age	0.98 (0.97-0.99)*	0.98 (0.97-0.99)*	0.99 (0.97-1.01)
Gender			
Male	1.73 (1.41-2.11)***	1.79 (1.34-2.38)***	1.80 (1.35-2.41)***
Social class			
High	1.72 (1.41-2.11)***	1.61 (1.23-2.09)***	1.60 (1.23-2.08)***
Employment status			
Employed	0.62 (0.49-0.79)***	0.76 (0.55-1.05)	0.76 (0.55-1.05)
Educational attainment			
College or University equivalent degree	0.28 (0.21-0.37)***	0.30 (0.23-0.41)***	0.27 (0.21-0.36)***
Graduate school	0.60 (0.43-0.83)**	0.60 (0.43-0.84)**	0.53 (0.38-0.73)***
Marital status			
Married	0.27 (0.20-0.36)***	0.27 (0.20-0.36)***	0.23 (0.17-0.30)***
Widowed/divorced/separated	0.02 (0.01-0.03)***	0.02 (0.01-0.03)***	0.01 (0.01-0.02)***
Smoking			
Yes	0.64 (0.47-0.89)**	0.63 (0.46-0.87)**	0.63 (0.46-0.87)**
Drinking			
Medium	1.59 (1.27-1.99)***	1.49 (1.19-1.86)***	1.39 (1.11-1.73)**
Heavy	0.43 (0.24-0.75)**	0.38 (0.25-0.57)***	0.40 (0.27-0.58)***
Network source	1.18 (1.06-1.31)**	1.16 (1.05-1.30)**	1.23 (1.11-1.37)***
Organizational participation			
High	2.03 (1.58-2.60)***	2.11 (1.67-2.68)***	2.55 (2.11-3.08)***
*Area-level variables*			
Concentrated disadvantage		0.90 (0.51, 1.57)	0.90 (0.58-1.38)
Organizational participation		0.58 (0.30-1.14)	0.67 (0.43-1.02)
*Cross-level interactions*			
Individual×area organizational participation			0.40 (0.32-0.50)***

*Random effects*			
Level2: Between-area variation (se)	0.174 (0.049)***	0.146 (0.039)***	0.154 (0.044)***
Intra-class correlation	0.050	0.042	0.044

*p < 0.05; **p < 0.01; ***p < 0.001.

All models adjusted for a dummy variable whether the respondents surveyed through a quota sampling, the within-area percentages of respondents surveyed through a quota sampling, and the response rates of area.

Since our sample combined two-survey data, we conducted a sensitivity test by conducting models with only first survey data (n = 3,898) to check the robustness of the results. Table 6 presents the result of multilevel analysis for self-reported health including only first-survey data. The ICC for the null model was 0.048 (data not shown). While there were changes in coefficients, the relationship between social capital variables and self-reported health remain as significant as in Model 3 with the same directions (Table 5). Additionally, there were slight changes in coefficients and statistically significant associations between control variables and self-reported health but with the same direction. The ICC for the full model was 0.030 which was lower than Model 3 (Table 5).

**Table 6:** Multilevel logistic analysis for self-reported health including only first-survey respondents (odds ratios and 95% confidence interval in parentheses; n = 3,898)

Fixed effects	Self-rated health (good/very good health)
	

*Individual level variables*	

Age	0.98 (0.97-1.00)
Gender	
Male	1.86 (0.24)***
Social class	
High	1.76 (1.39-2.22)***
Employment status	
Employed	0.75 (0.54-1.03)
Educational attainment	
College or University equivalent degree	0.27 (0.19-0.38)**
Graduate school	0.55 (0.37-0.81)**
Marital status	
Married	0.99 (0.70-1.41)
Widowed/divorced/separated	0.20 (0.05-0.75)*
Smoking	
Yes	0.81 (0.60-1.07)
Drinking	
Medium	1.07 (0.78-1.46)
Heavy	0.38 (0.24-0.61)***
Network source	1.22 (1.07-1.39)**
Organizational participation	
High	1.94 (1.50-2.53)***
*Area-level variables*	
Concentrated disadvantage	0.82 (0.52-1.30)
Organizational participation	0.86 (0.66-1.11)
*Cross-level interactions*	
Individual×area organizational participation	0.37 (0.28-0.48)***

*Random effects*	
Intra-class correlation null model	0.048
Intra-class correlation full model	0.030

*p < 0.05; **p < 0.01; ***p < 0.001.

The full model adjusted for the response rates of area.

## Discussion

Research aimed at explaining the association between social capital and health has been increasing, but several issues remain. Firstly, research of the topic has been sparsely conducted in non-Western countries. Secondly, studies using multilevel analysis suffer from a lack of contextual factors other than social capital. The aim of the present study was to examine the association between social capital and self-reported health using multilevel analysis in Seoul, South Korea to fill these gaps. It was aimed especially at examining whether individual or collective-level social capital are associated with self-reported health after controlling for possible confounders at both the individual and area-level using multilevel analysis in non-Western countries.

In terms of control variables, this study found significant associations at the individual-level. Men are more likely to report good/very good health than women. Respondents with a high perceived social class are more likely to report good/very good health than respondents with a low perceived social class. Compared to respondents with high school or below educational attainment, those with an educational attainment of a college or university equivalent degree and graduate school are less likely to report good/very good health. Respondents who are married or widowed/divorced/separated are less likely than single respondents to report good/very good health. Respondents who smoke are less likely to report better health than non-smokers. Compared to respondents who do not drink, medium drinkers are more likely to report better health, but heavy drinkers are less likely to report better health. However, we did not find any significant relationship at the area-level.

Previous studies have shown that educational attainment is positively associated with self-reported health [16,45,57]. Although our results differed from these studies, as educational attainment is negatively associated with self-reported health, several explanations are possible. According to Sen [58], self-reported health may be affected by individuals' experience, expectation, perception and education. Thus, it may be that well-educated individuals are more likely to show concern regarding their health, perceive illness, and have more expectations regarding their ideal health than less-educated individuals, as they are more likely to have interest and knowledge regarding health. This logic might be applied to South Korea where there is growing interests in physical well-being. Murray & Chen [59] also supported this idea by providing evidence of differences in self-reported morbidity rates in the United States and Indian states. They argue that one's knowledge (e.g., education) and experience (e.g., health service usage) may affect their own health ideals. And these factors are associated knowledge and perception of health.

Regarding the main interest of this study, this study found that individual-level social capital is associated with health. Both network resources and organizational participation which were considered individual-level social capital indicators are positively related to self-reported health. Respondents with higher levels of network resources are more likely to report good/very good health. And respondents with high organizational participation are more likely to report good/very good health than those with low organizational participation. There are several possible explanations for why social capital affects health. It is likely that people who have affluent network resources and participate in some organizations may have material and/or emotional support from others, which may buffer one's specific problem. Additionally, as previous studies showed, people with affluent social networks are less likely to experiences sadness and loneliness, which could improve one's health in turn. Participating in a certain organization exerts more effective social control over behaviors associated with bad health such as smoking and binge drinking, which is beneficial for one's overall [57]. Lastly, social capital may enhance a diffusion of health information and knowledge so people may adopt certain health related innovations which will increase one's health [26].

Although we did not find significant relationships between area-level organizational participation and self-reported health, we did find the cross-level interaction for social capital. Some studies [36,60] also found evidence of cross-level interaction between, micro-level and macro-level social capital variables. However, our results differ from these studies and are instead consistent with other studies [6,61]. Our results indicate that in areas with lower average organizational participation, individuals with high organizational participation are more likely to report better self-reported health than individuals with low organizational participation. However, as area-level organizational participation increases, this relationship is changed. In areas with higher organizational participation, high organizational participation individuals are less likely to report better self-reported health than low organizational participation individuals. This result is consistent with the previous argument that social capital may have not only positive but also negative effects [8]. According to Mansyur et al. [6], it is likely that individuals with high organizational participation may have more demands on their time and efforts in areas with high organizational participation. This may cause low organizational participation individuals to benefit from their participation in organizations. It is also likely that a "free riding" problem occurs, where individuals with low organizational participation may receive benefits from residing in areas with higher organizational participation, but they may spend less of their own time and efforts than high organizational participation individuals. This may cause less stress for low organizational participation individuals but more demands and stress for high organizational participation individuals [61].

In Asian countries, studies have been sparsely conducted to examine the association between social capital and health compared to the West. Since there are differences in the cultural and historical backgrounds between the Asian and Western countries, and the role of social capital is varied depending on the characteristics of societies [23,44], it remains questionable whether social capital is crucial to explain one's health status. Our results indicated that social capital, which was originated and developed in the Western countries, may be important for explaining health inequality in South Korea, at least at the individual-level. To our knowledge, no previous studies examining the association between social capital and health using multilevel analysis in South Korea could be found among peer-reviewed journals in English. Thus, more studies should be conducted in order to explicitly determine the association between social capital and health in South Korea.

Several limitations of the study should be recognized. First, the sample size of the areas in this study has potential problems. We used a relatively small sample size of 25 areas in Seoul, South Korea. We found the relationships between independent variables and self-reported health mainly at the individual-level in our final model. However, it is better to take large numbers of macro-units than a large number of micro-units for accuracy and higher power because the power of tests of higher-level effects depends more strongly on the number of macro-units than on the total sample size [35]. Therefore, it is likely that we did not find the contextual effects due to the smaller of sample sizes of the areas. However, given the fact that we found a cross-level interaction term, which needs larger groups than a multilevel model without an interaction term [62], this suggests that the sample size of macro level for the current study is not the only reason that causes the absence of are-level effects.

Second, the chosen spatial level may cause some problems. We cannot be sure that an administrative-area is the proper spatial scale for operating area-level social capital. Previous studies revealed that the contextual effects of social capital are varied depending on geographic level [16,44,63]. For reasons of practicality, the present study chose an administrative-area as an area-level, although other researchers might choose a different geographic level. Similarly, it is likely that the area-level confounder was not associated with self-reported health simply because it was not the proper spatial level. Thus, it is possible that a different spatial scale may cause different results from the current study. Additionally, it is a common practice to aggregate individual measures to higher levels of analyses, but we cannot be sure that this aggregate measure reflects the true contextual level of social capital [25]. Thus, Harpham et al. [64] argued that it is needed to use ecological social capital indicators such as voter turnout and the number of voluntary organizations per capita. Additional effort should be warranted to measure macro-level social capital properly.

Third, the data set for the current study is a cross-sectional sample. An important weakness of cross-sectional studies is that one cannot draw conclusions about the direction of the confirmed relationships, so the causality tends to be tentative. It is usually assumed that social capital effects self-reported health, but it may also be argued that self-reported health effects social capital. In order to resolve this problem, using a longitudinal study will provide a more concrete conclusion regarding the causality between social capital and health.

Fourth, this study was based on a questionnaire survey which relies on self-reporting. Compared to an objective measure, a subjective measure is less accurate. Also, the common method bias is more likely to occur.

Fifth, our sampling methods have several limitations. First, we combined two different surveys data which might cause biased results. However, we controlled for several factors to reduce the problem and confirmed that the main results were not changed markedly between analysis with full sample and only with the first survey sample. Second, the relatively low response rate of 43% may cause a selection bias. In order to reduce this problem, which might also cause significant ICC in self-reported health in the analysis with a full sample, we included the response rates of area in all multilevel models. Third, the sampling frame has a possible limitation and may not represent all Seoul citizens. It is known that a telephone directory has a household non-coverage problem, which threatens the representation of sample. Additionally, although we made callbacks three times, it is possible that calls were not reached simply because the targeted respondents were not at home at that time. Thus, respondents in this study may not represent Seoul citizens.

Finally, this study may suffer from omitted variable bias. Although we considered possible confounders at both the individual and area-levels, we may have omitted some variables which are possibly related to independent variables associated with self-reported health. For example, it is suggested that built environment is associated with social capital and health [65]. Thus, our inability to control for this factor may be confounding the association found in the study.

Despite these limitations, our study provides evidence that individual-level social capital is associated with self-reported health, even after controlling for both individual and area-level confounders. Both individual-level network resources and organizational participation are positively associated with self-reported health. Although area-level organizational participation was not significantly associated with self-reported health, this study found a cross-level interaction for individual and area-level organizational participation. Taken together, this study suggests, albeit tentatively, that policy makers should focus upon social capital when they make policies which aim to enhance one's health.

## Competing interests

The authors declare that they have no competing interests.

## Authors' contributions

SH made substantial contributions in designing, analyzing, interpreting data, writing, and revising the manuscript. HK acquired data, drafted, and wrote the manuscript. HL was involved in acquisition of data, supervised implementing of this study, and revising the manuscript critically. All authors read and agreed to the final draft of the manuscript.
